# Supplementary material for: Reversible structural evolution of sodium-rich rhombohedral Prussian blue for sodium-ion batteries
Source: Nat Commun. 2020 Feb 20;11:980. doi: 10.1038/s41467-020-14444-4 (PMC7033191; doi:10.1038/s41467-020-14444-4)
Supplement: Supplementary file 1 — Supplementary Information [file 41467_2020_14444_MOESM1_ESM.pdf]

Supplementary Information

Reversible structural evolution of sodium-rich rhombohedral Prussian blue for sodium-ion batteries

W. W., et al.

Supplementary Figures

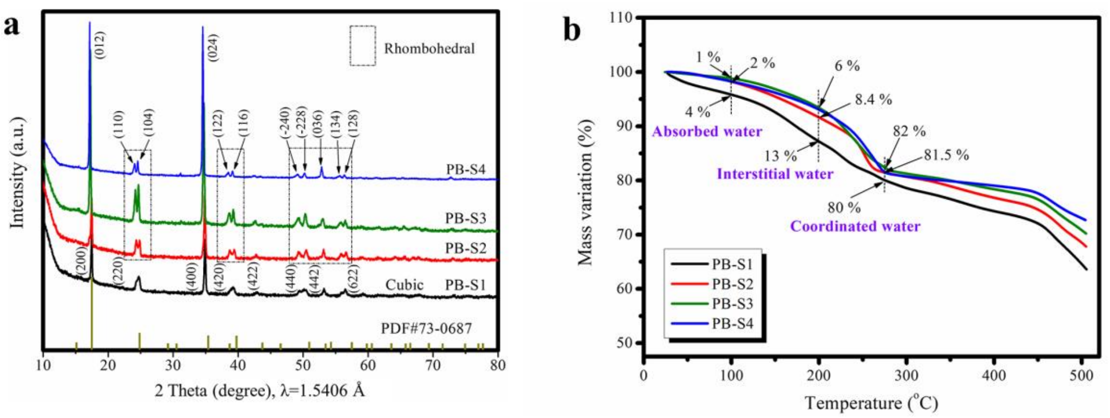

Supplementary Fig. 1 Characterizations of structure and water content of as-obtained $Na_{2-x}FeFe(CN)_6$ samples. (**a**) PXRD patterns and (**b**) TGA results for PB-S1, PB-S2, PB-S3 and PB-S4 samples.

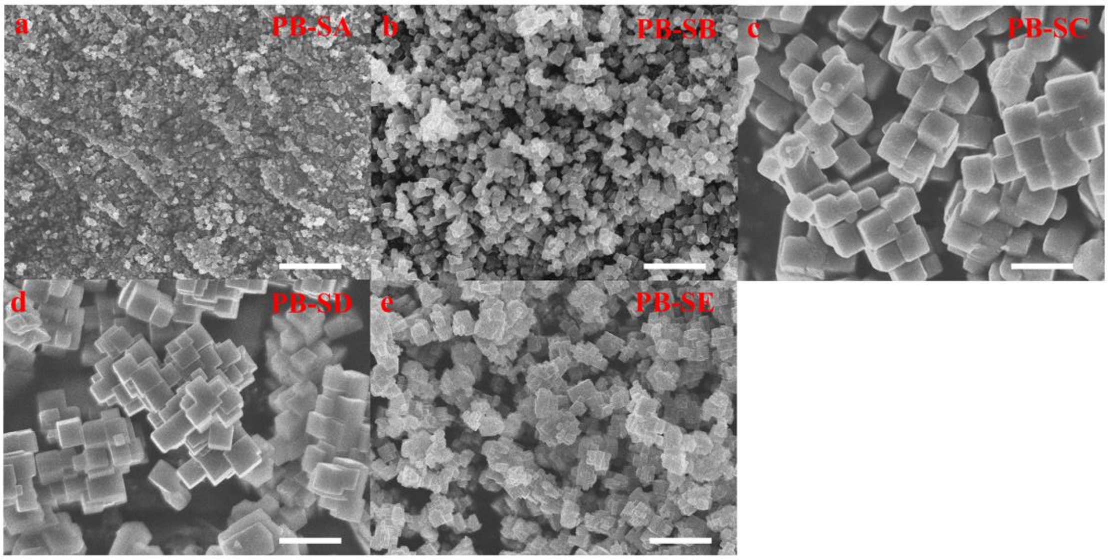

Supplementary Fig. 2 SEM images of as-obtained $Na_{2-x}FeFe(CN)_6$ samples: (**a**) PB-SA, (**b**) PB-SB, (**c**) PB-SC, (**d**) PB-SD, (**e**) PB-SE. Scale bars: 1 μm.

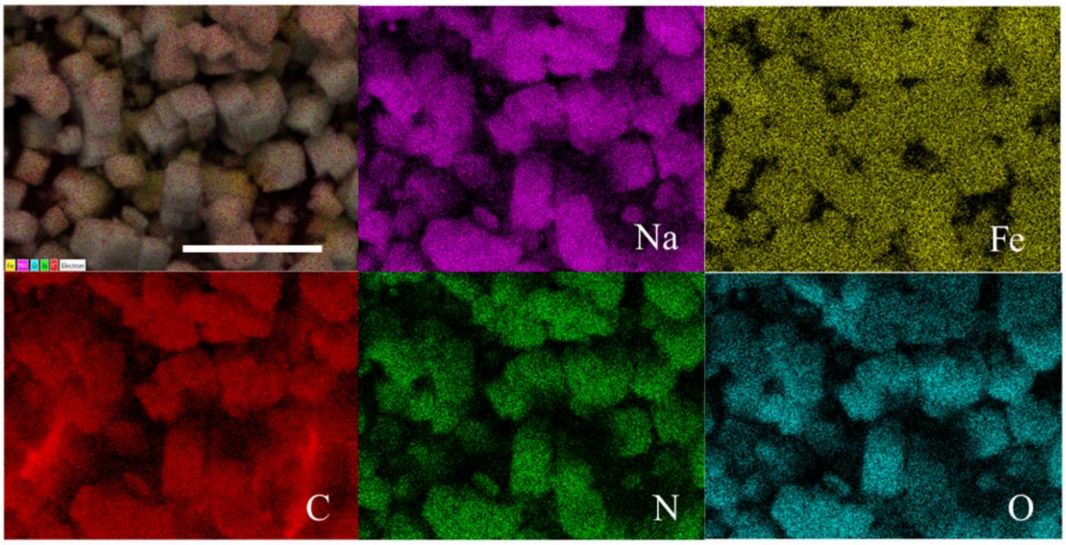

Supplementary Fig. 3 EDS mapping images of PB-S3 sample. Scale bar: 10 μm

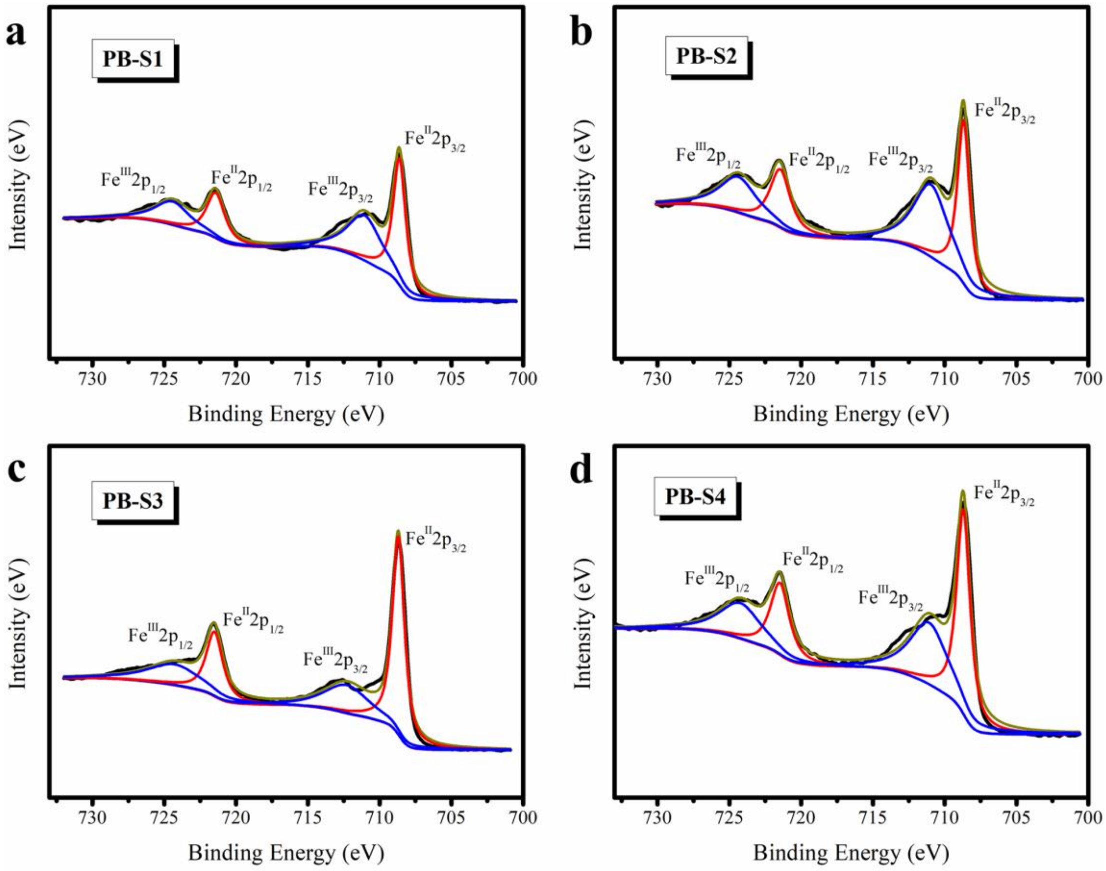

Supplementary Fig. 4 XPS spectra of Fe element for as-obtained $Na_{2-x}FeFe(CN)_6$ samples: (**a**) PB-S1, (**b**) PB-S2, (**c**) PB-S3 and (**d**) PB-S4.

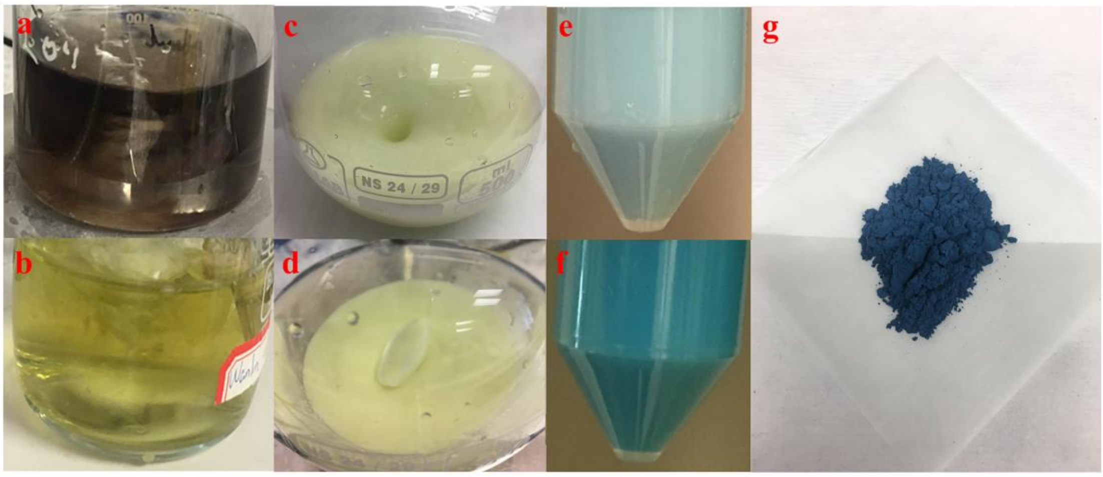

Supplementary Fig. 5 Digital photographs in precipitation process. $FeSO_4$ solution of (**a**) PB-SC sample without N_2 and (**b**) PB-S3 sample with N_2, the precipitation slurry of PB-S3 (**c**) before and (**d**) after aging for 1 hour; the final slurry of PB-S3 sample washed with (**e**) water 3 times and (**f**) ethanol 3 times, and the final dried powder of PB-S3 sample.

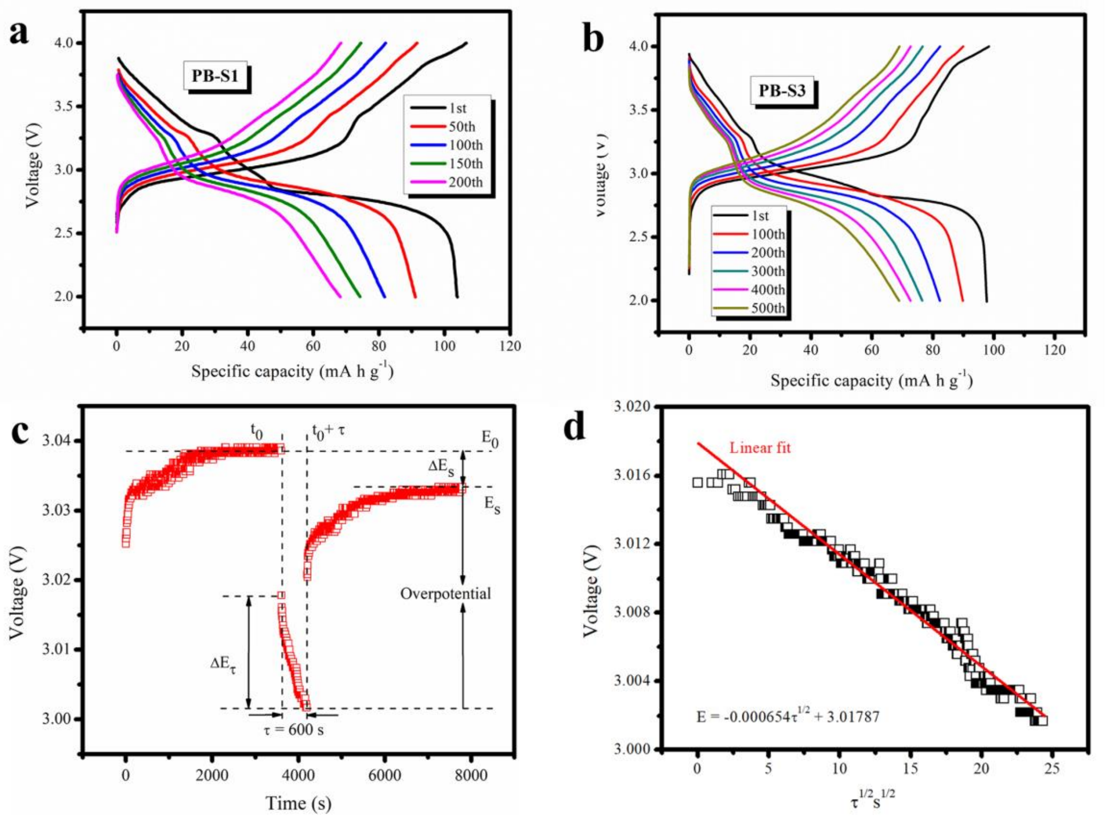

Supplementary Fig. 6 Electrochemical performances of PB-S1 and PB-S3 samples. Charge-discharge curves of (**a**) PB-S1 and (**b**) PB-S3 samples, an example of single step GITT (**c**) and (**d**) relationship between voltage and E vs. $\tau^{1/2}(s^{1/2})$ of PB-S3 sample.

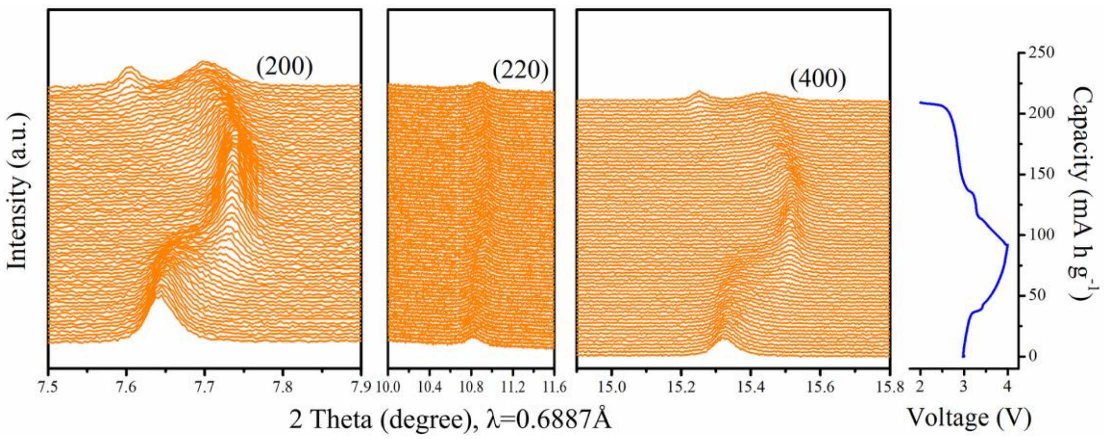

Supplementary Fig. 7 Investigation of structural change of PB-S1 sample during the first cycle. Synchrotron in-situ PXRD patterns of (200), (220) and (400) relections of cubic phase of PB-S1 sample during cycling.

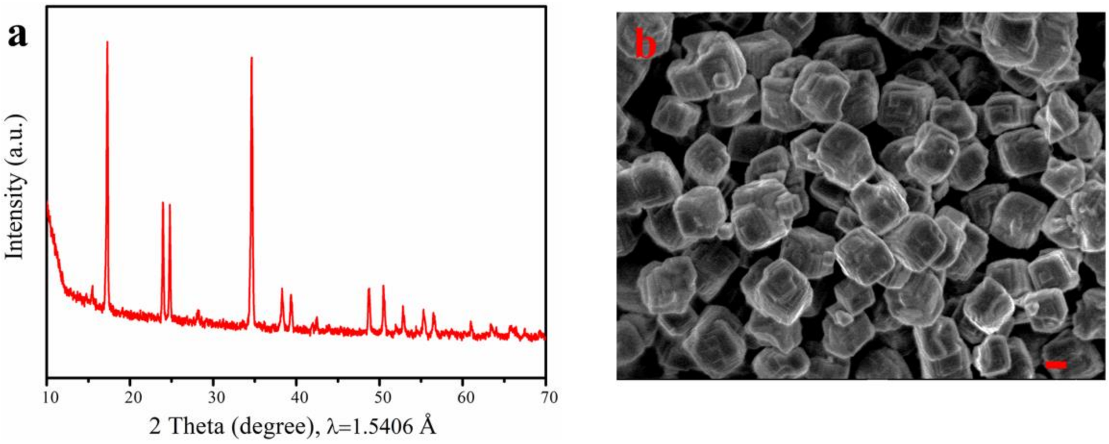

Supplementary Fig. 8 Characterization of structure and morphology of final product from 100 L reactor. (**a**) PXRD pattern and (**b**) SEM image of final product from 100 L reactor. Scale bar: 1 μm (**b**).

Supplementary Tables

Supplementary Table 1 Detailed structural information on the PB-S1 sample after Rietveld refinement.

PB-S1 ($Na_{1.53}Fe[Fe(CN)_6]\cdot4.2H_2O$). Cubic, space group *Fm-3m*, $a = b = c$ = 10.3711(1) Å, $\alpha = \beta = \gamma = 90^o$, V = 1115.5127(1) $Å^3$, weighted profile R-factor, R_{wp}=5.23 %					
Atom	x	y	z	Site	Occupation
Fe1	0.00000	0.00000	0.00000	4a	1
Fe2	0.50000	0.50000	0.50000	4b	1
C	0.1971(1)	0.00000	0.00000	24e	1
N	0.2888(1)	0.00000	0.00000	24e	1
Na	0.25000	0.25000	0.25000	8c	0.765(2)

Supplementary Table 2 Detailed structural information on the PB-S3 sample after Rietveld refinement.

PB-S3 ($Na_{1.73}Fe[Fe(CN)_6]\cdot3.8H_2O$). Rhombohedral, space group *R*-3, $a = b$ = 7.43079(1) Å, c = 17.6133(1) Å, $\alpha = \beta$ = 90º, γ = 120º, V = 842.2506(1) Å^3, weighted profile *R*-factor, R_{wp}=6.51 %					
Atom	x	y	z	Site	Occupation
Fe1	0.00000	0.00000	0.00000	3a	1
Fe2	0.33333	0.66667	0.16667	3b	1
C	0.1500(1)	0.2956(1)	0.0714(1)	18f	1
N	0.2001(1)	0.4121(1)	0.0997(1)	18f	1
Na	0.00000	0.00000	0.2323(1)	6c	0.86(2)

Supplementary Table 3 ICP results on Fe and Na for PB-S1, PB-S2, PB-S3 and PB-S4 samples.

Sample	Fe (ppm)	Na (ppm)
PB-S1	357200	112300
PB-S2	339400	116100
PB-S3	332400	118400
PB-S4	332700	112000

Supplementary Table 4 Comparison of electrochemical performances with other $Na_{2-x}FeFe(CN)_6$ reported by precipitation methods.

Sample	phase	Electrochemical performance	Reference
$Na_{1.7}FeFe(CN)_6$	Cubic	ICE=116 %, 129 mA h g^{-1} (77 % after 100 cycles)	[1]
$Na_{1.54}FeFe(CN)_6$	Rhombohedral	ICE=94.9 %, 108 mA h g^{-1} (48.1 % after 500 cycles)	[2]
Ppy-$Na_{1.54-x}FeFe(CN)_6$	Cubic	ICE=180 %, 108 mA h g^{-1} (79 % after 500 cycles)	[2]
Fe-HCF	Rhombohedral	ICE=92.1 %, 117.8 mA h g^{-1} (25.9 % after 800 cycles)	[3]
Fe-HCF@Ni-HCF	Cubic	ICE=189 %, 102 mA h g^{-1} (78 % after 800 cycles)	[3]
$Na_{1.73}Fe[Fe(CN)_6]_{0.98}$	Cubic	ICE=95.2 %, 123 mA h g^{-1} (73 % after 200 cycles)	[4]
PBA-3	Cubic	ICE=87 %, 90 mA h g^{-1} (86.3 % after 90 cycles)	[5]
$Na_{1.95}Fe[Fe(CN)_6]_{0.93}$	Rhombohedral	ICE=109 %, 130 mA h g^{-1} (78.8 % after 500 cycles)	[6]
$Na_{1.22}Fe[Fe(CN)_6]_{0.88}$	Cubic	ICE=89 %, 130 mA h g^{-1} (70 % after 50 cycles)	[7]
**$Na_{1.73}Fe[Fe(CN)_6]\cdot 3.8H_2O$**	**Rhombohedral**	**ICE=97.4 %, 100 mA h g^{-1} (71 % after 500 cycles)**	**This work**

Supplementary Reference

1. Liu, Y. et al. Sodium storage in Na-rich NaxFeFe$(CN)_6$ nanocubes. *Nano Energy* **12**, 386-393 (2015).
2. Tang, Y. et al. Polypyrrole-promoted superior cyclability and rate capability of $Na_xFe[Fe(CN)_6]$ cathodes for sodium-ion batteries. *J. Mater. Chem. A* **4**, 6036-6041 (2016).
3. Wan, M. et al. Core-shell hexacyanoferrate for superior Na-ion batteries. *J. Power Sources* **329**, 290-296 (2016).
4. Yang, Y., et al. Influence of Structural Imperfection on Electrochemical Behavior of Prussian Blue Cathode Materials for Sodium Ion Batteries. *J. Electrochem. Soc.* **163**, A2117-A2123 (2016).
5. Fu, H., et al. Enhanced storage of sodium ions in Prussian blue cathode material through nickel doping. *J. Mater. Chem. A* **5**, 9604-9610 (2017).
6. Huang, Y., et al. A novel border-rich Prussian blue synthetized by inhibitor control as cathode for sodium ion batteries. *Nano Energy* **39**, 273-283 (2017).
7. Bie, X., et al. Synthesis and electrochemical properties of Na-rich Prussian blue analogues containing Mn, Fe, Co, and Fe for Na-ion batteries. *J. Power Sources* **378**, 322-330 (2018).
